# miR-143 and miR-145 synergistically regulate ERBB3 to suppress cell proliferation and invasion in breast cancer

**DOI:** 10.1186/1476-4598-13-220

**Published:** 2014-09-24

**Authors:** Xin Yan, Xi Chen, Hongwei Liang, Ting Deng, Weixu Chen, Suyang Zhang, Minghui Liu, Xiujuan Gao, Yanqing Liu, Chihao Zhao, Xueliang Wang, Nan Wang, Jialu Li, Rui Liu, Ke Zen, Chen-Yu Zhang, Baorui Liu, Yi Ba

**Affiliations:** Department of Gastrointestinal Medical Oncology, National Clinical Research Center of Cancer, Key Laboratory of Cancer Prevention and Therapy of Tianjin, Tianjin Medical University Cancer Institute and Hospital, Huanhuxi Road, Tiyuanbei, Tianjin 300060 China; Jiangsu Engineering Research Center for microRNA Biology and Biotechnology, State Key Laboratory of Pharmaceutical Biotechnology, School of Life Sciences, Nanjing University, 22 Hankou Road, Nanjing, 210093 China; The Comprehensive Cancer Center of Drum Tower Hospital, Medical School of Nanjing University and Clinical Cancer Institute of Nanjing University, Nanjing, Jiangsu 210008 P.R. China; First Affiliated Hospital of Nanjing Medical University, Nanjing, 210009 China

**Keywords:** microRNA, miR-143, miR-145, ERBB3, Breast cancer, Proliferation, Invasion

## Abstract

**Introduction:**

ERBB3, one of the four members of the ErbB family of receptor tyrosine kinases, plays an important role in breast cancer etiology and progression. In the present study, we aimed to identify novel miRNAs that can potentially target ERBB3 and their biological functions.

**Method:**

The expression levels of miR-143/145 and target mRNA were examined by relative quantification RT-PCR, and the expression levels of target protein were detected by Western blot. We used bioinformatic analyses to search for miRNAs that can potentially target ERBB3. Luciferase reporter plasmids were constructed to confirm direct targeting. Furthermore, the biological consequences of the targeting of ERBB3 by miR-143/145 were examined by cell proliferation and invasion assays in vitro and by the mouse xenograft tumor model in vivo.

**Results:**

We identified an inverse correlation between miR-143/145 levels and ERBB3 protein levels, but not between miR-143/145 levels and ERBB3 mRNA levels, in breast cancer tissue samples. We identified specific targeting sites for miR-143 and miR-145 (miR-143/145) in the 3’-untranslated region (3’-UTR) of the ERBB3 gene and regulate ERBB3 expression. We demonstrated that the repression of ERBB3 by miR-143/145 suppressed the proliferation and invasion of breast cancer cells, and that miR-143/145 showed an anti-tumor effect by negatively regulating ERBB3 in the xenograft mouse model. Interestingly, miR-143 and miR-145 showed a cooperative repression of ERBB3 expression and cell proliferation and invasion in breast cancer cells, such that the effects of the two miRNAs were greater than with either miR-143 or miR-145 alone.

**Conclusion:**

Taken together, our findings provide the first clues regarding the role of the miR-143/145 cluster as a tumor suppressor in breast cancer through the inhibition of ERBB3 translation. These results also support the idea that different miRNAs in a cluster can synergistically repress a given target mRNA.

**Electronic supplementary material:**

The online version of this article (doi:10.1186/1476-4598-13-220) contains supplementary material, which is available to authorized users.

## Introduction

Breast cancer is the most common cancer and principle cause of death from cancer in women worldwide [1, 2]. The overexpression of the ErbB family of receptor tyrosine kinases (RTKs) is thought to be important in the development of breast cancer [3, 4]. The ErbB family consists of four closely related members, including ERBB1/EGFR, ERBB2/HER2, ERBB3/HER3 and ERBB4/HER4 [3, 4]. Ligand binding to the extracellular domain of ErbB receptors results in the formation of homodimers or heterodimers. Receptor dimerization is activated by the intrinsic tyrosine kinase-mediated autophosphorylation of the receptors within the cytoplasmic domain, and the phosphorylation results in the recruitment of downstream effector proteins that activate multiple signaling pathways, including the mitogen-activated protein kinase (MAPK) and phosphoinositide 3-kinase (PI3K) pathways [3, 4]. Through these molecular mechanisms, the ErbB family regulates the proliferation, differentiation and survival of human breast epithelial cells, and aberrant expression or activity of this family has been strongly linked to the etiology of breast cancer [3, 4].

For many years, it was believed that the ERBB2 receptor played a major role in the development and progression of breast malignancies [5–7]. ERBB2 is a therapeutic target in breast tumors that overexpress the receptor. However, an increasingly important role for the ERBB3 receptor in the genesis of breast cancer has emerged. ERBB3 is frequently upregulated in breast cancer, and overexpression of ERBB3 is positively associated with metastasis, histological grade, tumor size and recurrence [5–7]. ERBB3 has also been implicated in the development of resistance to anti-estrogens and ErbB tyrosine kinase inhibitors, such as tamoxifen and gefitinib, respectively [5–7]. ERBB3 is distinct in that it is the only member of the ErbB receptor family that lacks tyrosine kinase activity. In this way, activation of this receptor is achieved only through the formation of heterodimers with EGFR, ERBB2 or ERBB4 [5–7]. Consistent with this idea, the ERBB2/3 heterodimer has been demonstrated to be an “oncogenic unit” and a key factor in the proliferation and invasion of human breast cancer cells. Furthermore, co-expression of ERBB2 and ERBB3 is significantly associated with decreased patient survival [5–8]. ERBB3 is also unique among the ErbB family in its ability to directly recruit and activate the PI3K/Akt signaling pathway, which undoubtedly favors tumor growth and progression [5–7].

Over the past decade, a novel class of small RNA molecules known as microRNAs (miRNAs) has emerged as a major regulator of the initiation and progression of human cancers, including breast cancer [9, 10]. miRNAs are small (~22-nucleotide long), single-stranded noncoding RNA molecules that negatively regulate gene expression by binding to the 3’-untranslated region (3’-UTR) of target mRNA molecules, which results in either degradation of the transcript or inhibition of translational [11–13]. Many miRNAs work in conjunction with one another to fine tune protein expression on a global level. Thus, miRNAs play a significant role in post-transcriptional gene regulation. Importantly, recent studies indicate that the dysregulation of miRNAs is associated with human malignancies and suggest a causal role of miRNAs in cancer etiology. miRNAs presumably have a role in cancer because they can affect the translation and stability of targeted oncogenes or tumor suppressors, which eventually influence cellular physiology [14–16]. For example, miR-143 and miR-145 (miR-143/145), which are located in a cluster within the 5q32-33 chromosomal region, are downregulated in many types of cancers, including colon cancer and breast cancer [17, 18]. miR-143 and miR-145 have been shown to possess anti-tumorigenic activity, with involvement in various cancer-related events such as proliferation, invasion and migration. The target genes of miR-143 identified and verified thus far are primarily MAPK signaling molecules, such as ERK5 and KRAS [19, 20]. Target genes of miR-145 include Mucin-1 [21], RTKN [22], c-Myc [23] and EGFR [24].

Although dysregulation of ERBB3 and miRNAs is associated with tumorigenesis in human breast cancer, little is known about the natural miRNAs that act on ERBB3. In this study, we found that ERBB3 protein levels, but not mRNA levels, were upregulated in breast cancer tissues in comparison to adjacent noncancerous tissues. We hypothesized that the high levels of ERBB3 are a consequence of miRNA regulation, and we subsequently identified a cluster of miRNAs, including miR-143 and miR-145, that directly target and regulate the expression of ERBB3 protein in breast cancer cells. Furthermore, we showed that miR-143/145 inhibited ERBB3 expression and consequently suppressed the proliferation and invasion of breast cancer cells.

## Materials and methods

### Human tissue

Breast cancer tissues and paired adjacent noncancerous tissues were derived from patients undergoing a surgical procedure at the Tianjin Medical University Cancer Institute and Hospital (Tianjin, China). Both tumors and noncancerous tissues were confirmed histologically. The pathological type of each cancer was determined to be infiltrating ductal carcinoma. Written consent was provided by all of the patients, and the Ethics Committee of Tianjin Medical University Cancer Institute and Hospital approved all aspects of this study. Tissue fragments were immediately frozen in liquid nitrogen at the time of surgery and stored at −80°C. The clinical features of the patients are listed in Additional file 1: Table S1.

### Cell culture

The human breast cancer cell line MCF-7 and MBA-MD-231 were purchased from the Shanghai Institute of Cell Biology, Chinese Academy of Sciences (Shanghai, China). MCF-7 and MBA-MD-231 cells were cultured in DMEM (Gibco, Carlsbad, CA, USA) supplemented with 10% fetal bovine serum (Gibco) in a humidified incubator at 37°C with 5% CO_2_.

### RNA isolation and quantitative RT-PCR

Total RNA was extracted from the cultured cells and tissues using TRIzol Reagent (Invitrogen) according to the manufacturer’s instructions. Assays to quantify mature miRNAs were performed using Taqman microRNA probes (Applied Biosystems, Foster City, CA) according to the manufacturer’s instructions. Briefly, 1 μg of total RNA was reverse-transcribed to cDNA using AMV reverse transcriptase (TaKaRa, Dalian, China) and a stem-loop RT primer (Applied Biosystems). The reaction conditions were as follows: 16°C for 30 min, 42°C for 30 min and 85°C for 5 min. Real-time PCR was performed using a TaqMan PCR kit and an Applied Biosystems 7300 Sequence Detection System (Applied Biosystems). The reactions were incubated in a 96-well optical plate at 95°C for 10 min, followed by 40 cycles of 95°C for 15 s and 60°C for 1 min. All of the reactions were run in triplicate. After the reactions were complete, the cycle threshold (C_T_) data were determined using fixed threshold settings, and the mean CT was determined from triplicate PCRs. A comparative C_T_ method was used to compare each condition to the control reactions. U6 snRNA was used as an internal control, and the relative amount of miRNA normalized to U6 was calculated with the equation 2^-ΔΔCT^, in which ΔΔC_T_ = (C_T miRNA_ − C_T U6_)_target_ − (C_T miRNA_ − C_T U6_)_control_.

To quantify ERBB3 and β-actin mRNA, 1 μg of total RNA was reverse-transcribed to cDNA using Oligo d(T)18 primers (TaKaRa) and ThermoScript reverse transcriptase (Invitrogen). The reaction conditions were as follows: 42°C for 60 min and 70°C for 10 min. Real-time PCR was then performed with the RT product, and these reactions included SYBR Green dye (Invitrogen) and specific primers for ERBB3 and β-actin. The sequences of the primers were as follows: ERBB3 (sense): 5’-TTCCGAGATGGGCAACTCTC-3’; ERBB3 (antisense): 5’-CTTGCAGACTTCGTGACAGG-3’; β-actin (sense): 5’-AGGGAAATCGTGCGTGAC-3’; and β-actin (antisense): 5’-CGCTCATTGCCGATAGTG-3’. The reactions were incubated at 95°C for 5 min, followed by 40 cycles of 95°C for 30 s, 60°C for 30 s and 72°C for 30 s. After the reactions were complete, the C_T_ values were determined by setting a fixed threshold. The relative amount of ERBB3 mRNA was normalized to β-actin.

### miRNA overexpression or knockdown

miRNA overexpression was achieved by transfecting cells with a miRNA mimic (a synthetic RNA oligonucleotide duplex mimicking miRNA precursor), whereas knockdown was achieved by transfecting cells with a miRNA inhibitor (a chemically modified single-stranded antisense oligonucleotide designed to specifically target mature miRNA). Synthetic RNA molecules, including pre-miR-143, pre-miR-145, anti-miR-143, anti-miR-145 and scrambled negative control RNA (pre-miR-control and anti-miR-control), were purchased from GenePharma (Shanghai, China). MCF-7 cells were seeded in 6-well plates and transfected with Lipofectamine 2000 (Invitrogen) on the following day when the cells were approximately 70% confluent. For overexpression of miRNAs, 100 pmol of pre-miR-143, 100 pmol of pre-miR-145 or 50 pmol of both pre-miR-143 and pre-miR-145 were used. For knockdown of miRNAs, 100 pmol of anti-miR-143, 100 pmol of anti-miR-145 or 50 pmol of both anti-miR-143 and anti-miR-145 were used. After 6 h, the medium was changed to DMEM that was supplemented with 2% fetal bovine serum. The cells were harvested 24 h or 48 h after the transfection for the isolation of total RNA or protein, respectively.

### Plasmid construction and siRNA interference assay

A mammalian expression plasmid (pReceiver-M02-ERBB3) designed to specially express the full-length open reading frame (ORF) of human ERBB3 without the miR-143/145–responsive 3’-UTR was purchased from GeneCopoeia (Germantown, MD, USA). An empty plasmid served as a negative control. The siRNA (sequence: AAGAGCGACTAGACATCAAGC) targeting human ERBB3 was designed and synthesized by Invitrogen (Carlsbad, CA, USA). A scrambled siRNA (Invitrogen) was included as a negative control. Overexpression plasmid or siRNA were transfected into MCF-7 cells using Lipofectamine 2000 (Invitrogen) according to the manufacturer’s instructions. Total RNA or protein was isolated 24 h or 48 h after transfection. The ERBB3 mRNA and protein expression levels were assessed by quantitative RT-PCR and western blotting.

### Luciferase reporter assay

The entire 3’-UTR of human ERBB3 was amplified by PCR using human genomic DNA as a template. The PCR products were inserted into the p-MIR-reporter plasmid (Ambion, Austin, TX, USA). The insertion was confirmed to be correct by DNA sequencing. To test the binding specificity, the sequences that interact with the seed sequence of miR-143 and miR-145 were mutated (from UUGGGAG to AACCCUC for the miR-143 binding site; and from UUGGGAG to AACCCUC for the miR-145 binding site), and the mutant ERBB3 3’-UTR was inserted into an equivalent luciferase reporter plasmid. For luciferase reporter assays, MCF-7 cells were seeded in 6-well plates and co-transfected with 2 μg of firefly luciferase reporter plasmid, 2 μg of β-galactosidase (β-gal) expression plasmid (Ambion), and equal amounts (100 pmol) of miR-143/145 mimic, inhibitor, or scrambled negative control RNA using Lipofectamine 2000 (Invitrogen). The β-gal plasmid was used as a transfection control. Cells were harvested 24 h after transfection and analyzed for luciferase activity using a luciferase assay kit (Promega, Madison, WI, USA).

### Protein isolation and Western blotting

Cells or tissues were lysed in a RIPA lysis buffer (50 mM Tris–HCl, 150 mM NaCl, 0.1% SDS, 1% NP-40, 0.25% sodium deoxycholate and 1 mM EDTA, pH 8.0) with freshly added protease inhibitor cocktail (Roche) for 30 min on ice and were then centrifuged at 16,000 × g at 4°C for 10 min. The supernatant was collected, and the protein concentration was calculated with a BCA protein assay kit (Thermo Scientific, Rockford, IL, USA). Proteins were separated by SDS-PAGE (Bio-Rad). After electrophoresis, the proteins were electrotransferred to PVDF membranes (Bio-Rad) and then blocked with 5% skim milk for 1 h. The membranes were then incubated with primary antibodies at 4°C for 12 h. After three washes in TBST, the membranes were incubated with horseradish peroxidase-conjugated secondary antibody for 1 h at room temperature. After three washes, the membranes were incubated with the SuperSignal West Pico chemiluminescence substrate (Pierce). The protein levels were normalized by probing the same blots with α-Tubulin antibody. Anti-ERBB3 and anti-α-Tubulin antibodies were purchased from Abcam (ab34641; Cambridge, MA) and Santa Cruz Biotechnology (B-7; CA, USA), respectively.

### Construction of miR-143/145 overexpression lentiviral vector

A 300-bp fragment containing genomic miR-143 and miR-145 sequences was generated by PCR amplification from human DNA and subsequently cloned into the Lenti-PacTM human immunodeficiency virus (HIV) expression packing system (Invitrogen). miR-143/145 overexpression lentiviral vector was added to MCF-7 cells at 70% confluence in 6-well plates or 10-cm dishes at an MOI of 10 together with polybrene at a final concentration of 5 μg/mL according to the manufacturer’s instructions. Cells were then harvested for quantitative RT-PCR, Western blotting or animal experiments.

### Cell proliferation assay

MCF-7 cells were plated at 5 × 10^4^ cells per well in 96-well plates and then incubated overnight in DMEM supplemented with 10% FBS. Cells were collected 12, 24, 36, 48 and 60 hours post-transfection. After transfection, 20 μL of 3-(4,5-dimethylthiazol-2-yl)-2,5-diphenyl tetrazolium bromide (MTT) (5 mg/mL) was added into a corresponding test well and incubated for 4 h. The supernatant was then discarded, and 150 μL of DMSO was added to each well to dissolve the precipitate. Absorbance was measured at a wavelength of 490 nm.

### Transwell invasion assay

Transwell invasion assays were performed using Matrigel Invasion Chambers (BD Biosciences, Bedford, MA) with inserts containing an 8-μm pore-sized membrane with a thin layer of Matrigel. MCF-7 cells were transfected and harvested as mentioned above and were seeded at a density of 5 × 10^6^/well on the upper chamber with serum-free DMEM. DMEM with 10% FBS was put into the lower compartment as chemo-attractant. Cells were allowed to invade for 48 hours. Remaining cells in the upper chamber were scraped out with a cotton swap. Matrigel membranes were fixed with ice-cold methanol and then stained with 0.1% crystal violet solution. The invaded cells were counted by microscopic examination. Each experiment was performed in triplicate and repeated twice.

### Establishment of tumor xenografts in mice

Four-week-old male C57BL/6 J mice were purchased from the Model Animal Research Center of Nanjing University (Nanjing, China) and maintained under specific pathogen-free conditions at Nanjing University. Untreated MCF-7 cells or MCF-7 cells infected with the miR-143/145 overexpression lentiviral vector or transfected with the ERBB3 expression plasmid were injected subcutaneously into C57BL/6 J mice (1 × 10^7^ cells per mouse, 7 mice per group). Mice were sacrificed after one month. After the tumors were separated from the animals, the length, width and height of the tumors was measured with digital calipers. The tumor weights were then determined and the ellipsoid volume was calculated using the following formula: Volume = π/6 × (length) × (width) × (height). All animal care and handling procedures were performed in accordance with the National Institutes of Health’s Guide for the Care and Use of Laboratory Animals and were approved by the Institutional Review Board of Nanjing University (Nanjing, China).

### Statistical analysis

All of the Western blot and Transwell invasion assay images are representative of at least three independent experiments. Quantitative RT-PCR, the luciferase reporter assay and the cell viability assay were performed in triplicate, and each experiment was repeated several times. The results are presented as the means ± SE of at least three independent experiments. Statistical analysis was performed using a Student’s t-test, and the significance level was set as p < 0.05.

## Results

### Upregulation of ERBB3 protein but not mRNA in breast cancer tissues

**Figure 1:**
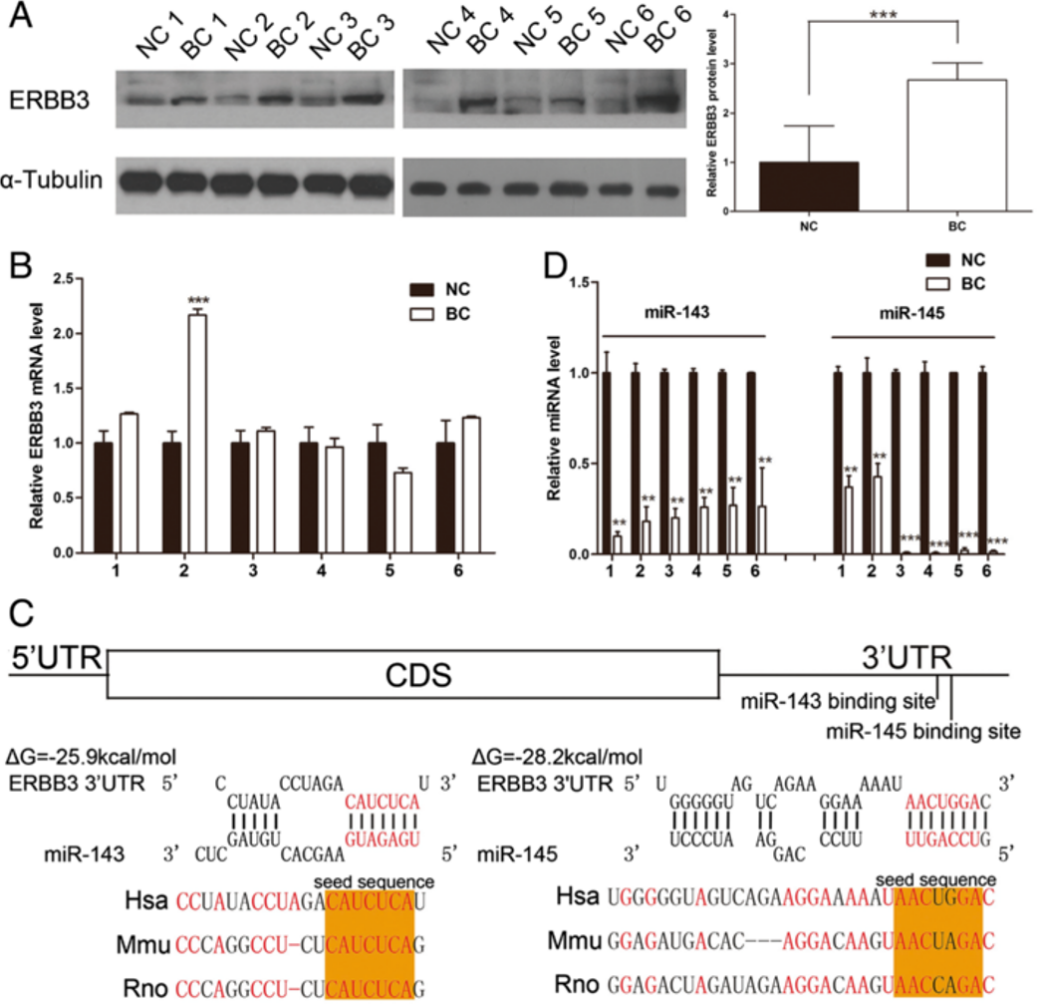
**Inverse correlation between miR-143/145 levels and ERBB3 protein levels in human breast cancer tissues. (A)** Western blot analysis of ERBB3 protein levels in six pairs of breast cancer tissue (BC) and noncancerous tissue (NC) samples. Left panel: representative image; right panel: quantitative analysis. **(B)** Quantitative RT-PCR analysis of ERBB3 mRNA levels in six pairs of BC and NC samples. **(C)** Quantitative RT-PCR analysis of miR-143 and miR-145 levels in six pairs of BC and NC samples. **(D)** Schematic description of the hypothetical duplexes formed by the interactions between the binding sites in the ERBB3 3’-UTR (top) and miR-143/145 (bottom). The predicted free energy value of each hybrid is indicated. The seed recognition sites are denoted, and all nucleotides in these regions are highly conserved across species, including human, mouse and rat. * P < 0.05; ** P < 0.01; *** P < 0.001.

We first determined the expression patterns of ERBB3 in human breast cancer tissues. After measuring the protein levels of ERBB3 in six pairs of breast cancer tissues and corresponding noncancerous tissues, we found that ERBB3 protein levels were dramatically higher in the breast cancer tissues (Figure 1A). However, although ERBB3 protein was consistently upregulated in breast cancer, ERBB3 mRNA levels did not differ significantly between the cancer and noncancerous tissues (Figure 1B). This disparity between protein and mRNA in ERBB3 expression in breast cancers strongly suggests that a post-transcriptional mechanism is involved in ERBB3 regulation.

### Identification of conserved miR-143 and miR-145 target sites within the 3’-UTR of ERBB3

One important mode of post-transcriptional regulation is the repression of mRNA transcripts by miRNAs. miRNAs are therefore likely to play a biologically relevant role in regulating ERBB3 expression in breast cancer. Three computational algorithms, including TargetScan [25], miRanda [26] and PicTar [27], were used in combination to identify potential miRNAs that can target ERBB3. Using these approaches, miR-143 and miR-145 were identified as candidate miRNAs. miR-143 and miR-145 are two miRNAs that are located within the same cluster, but they do not share sequence homology. Furthermore, all three algorithms predicted that these miRNAs are regulators of ERBB3. miR-143 and miR-145 were thus selected for further experimental verification. Interest in the miR-143/145 cluster was also supported by the recent identification of these two miRNAs as candidate breast cancer suppressors [9]. The predicted interactions between miR-143/145 and the targeting sites within the 3’-UTR of ERBB3 are illustrated in Figure 1C. One predicted hybridization was observed between both miR-143 and miR-145 and the 3’-UTR of ERBB3. Though the two targeting sites within the 3’-UTR of ERBB3 were nearby one another, the sites were non-overlapping. The minimum free energy values of the hybridizations between miR-143 and ERBB3 and between miR-145 and ERBB3 were −25.9 and −28.2 kcal/mol, respectively, which are well within the range of genuine miRNA-target pairs. Moreover, there was perfect base-pairing between the seed region (the core sequence that encompasses the first 2–8 bases of the mature miRNA) and the cognate targets. Furthermore, the miR-143/145 binding sequences in the ERBB3 3’-UTR were highly conserved across species.

### Detection of an inverse correlation between miR-143/143 and ERBB3 levels in breast cancer tissues

miRNAs are generally thought to have expression patterns that are opposite to that of their targets [11–13]. We next investigated whether miR-143/145 were inversely correlated with ERBB3 in breast cancer. After determining the levels of miR-143 and miR-145 in the same six pairs of breast cancer tissues and corresponding noncancerous tissues, we showed that miR-143 and miR-145 levels were indeed downregulated in breast cancer tissues (Figure 1D). Thus, ERBB3 was determined to be a miR-143/145 target, based on both computational predictions and the inverse correlation between miR-143/145 levels and ERBB3 protein levels, but not mRNA levels, in human breast cancer.

### Validation of ERBB3 as a direct target of miR-143/145

**Figure 2:**
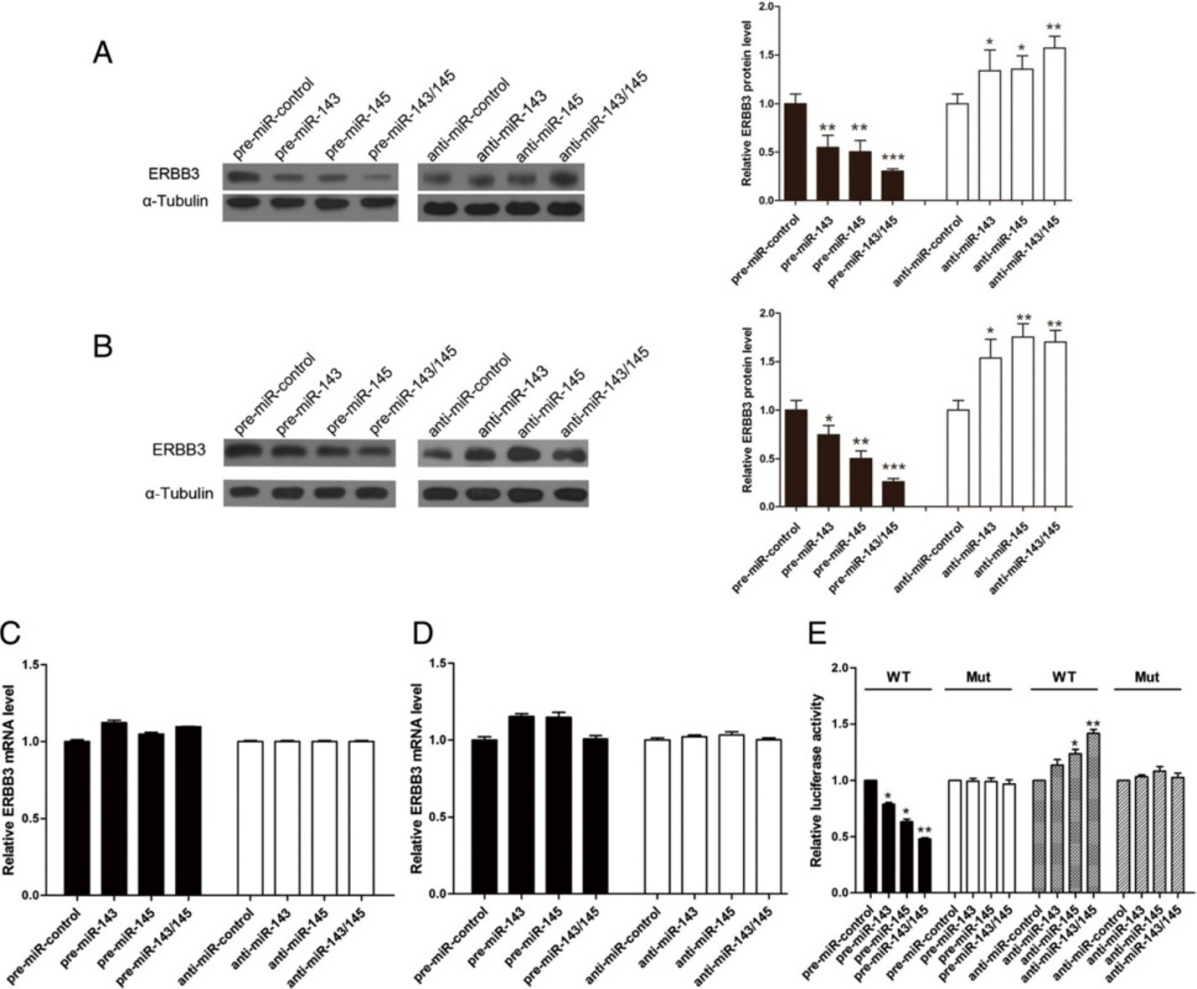
**miR-143/145 directly regulate ERBB3 expression at the post-transcriptional level. (A and B)** Western blot analysis of ERBB3 protein levels in MCF-7 **(A)** and MBA-MD-231 **(B)** cells treated with pre-miR-control, pre-miR-143, pre-miR-145 or both pre-miR-143 and pre-miR-145 and in cells treated with anti-miR-control, anti-miR-143, anti-miR-145 or both anti-miR-143 and anti-miR-145. Left panel: representative image; right panel: quantitative analysis. **(C and D)** Quantitative RT-PCR analysis of ERBB3 mRNA levels in MCF-7 **(C)** and MBA-MD-231 **(D)** cells treated with pre-miR-control, pre-miR-143, pre-miR-145 or both pre-miR-143 and pre-miR-145 and in cells treated with anti-miR-control, anti-miR-143, anti-miR-145 or both anti-miR-143 and anti-miR-145. **(E)** Direct recognition of the ERBB3 3’-UTR by miR-143/145. MCF-7 cells were co-transfected with firefly luciferase reporters containing either wild-type (WT) or mutant (Mut) miR-143/145 binding sites in the ERBB3 3’-UTR and pre-miR-control, pre-miR-143, pre-miR-145 or both pre-miR-143 and pre-miR-145, or with anti-miR-control, anti-miR-143, anti-miR-145 or both anti-miR-143 and anti-miR-145. Twenty-four hours after transfection, the cells were assayed using a luciferase assay kit. The results are displayed as the ratio of firefly luciferase activity in the miR-143/145-transfected cells to the activity in the control cells. * P < 0.05; ** P < 0.01.

The correlation between miR-143/145 and ERBB3 was further examined by evaluating ERBB3 expression in the human breast cancer cell line MCF-7 and MBA-MD-231 after overexpression or knockdown of miR-143/145. In these experiments, overexpression was achieved by transfecting cells with pre-miR-143 and/or pre-miR-145, which are synthetic RNA oligonucleotides that mimic the miR-143 and miR-145 precursors. Knockdown of miR-143/145 was achieved by transfecting cells with anti-miR-143 and/or anti-miR-145, which are chemically modified antisense oligonucleotides that are designed to specifically target mature miR-143 and miR-145. The efficient overexpression and knockdown of miR-143/145 in MCF-7 cells are shown in Additional file 1: Figure S1A and B. Cellular miR-143/145 levels were increased approximately 400-fold when MCF-7 cells were transfected with pre-miR-143/145, and these levels dropped to approximately 20% of the normal level when MCF-7 cells were treated with anti-miR-143/145. As anticipated, overexpression of miR-143 or miR-145 significantly suppressed ERBB3 protein levels in MCF-7 cells (Figure 2A). Interestingly, co-treatment of cells with both pre-miR-143 and pre-miR-145 enhanced the suppressive effect on ERBB3 protein expression compared to treatments with either pre-miR-143 or pre-miR-145 alone (Figure 2A). These results indicated that the suppressive effects of miR-143 and miR-145 on ERBB3 protein expression was not an individual response but was instead a synergistic effect. Furthermore, the expression of ERBB3 protein was significantly increased in MCF-7 cells transfected with anti-miR-143 or anti-miR-145, and the greatest increase in expression occurred when both anti-miR-143 and anti-miR-145 were used simultaneously (Figure 2A). These results further supported a synergistic effect of miR-143 and miR-145 on ERBB3 downregulation in MCF-7 cells. To demonstrate the robust of the test, we repeated the above experiments in additional breast cancer cell line, MBA-MD-231. miR-143/145 levels were significantly increased when MBA-MD-231 cells were transfected with pre-miR-143/145, but their levels were unchanged when MBA-MD-231 cells were treated with anti-miR-143/145 (Additional file 1: Figure S1C and D), which may be due to the low expression level of miR-143/145 in MBA-MD-231 cells. Consistent with the cellular levels of miR-143/145 after transfection, ERBB3 protein levels were downregulated when MBA-MD-231 cells were treated with pre-miR-143/145, but their levels were unaffected by treatment with anti-miR-143/145 (Figure 2B). To determine the regulatory level at which miR-143/145 influenced ERBB3 expression, we repeated the above experiments and examined the expression of ERBB3 mRNA after transfection. Overexpression or knockdown of miR-143/145 did not affect ERBB3 mRNA stability in MCF-7 and MBA-MD-231 cells (Figure 2C and D). These results demonstrated that miR-143/145 specifically regulate ERBB3 expression at the post-transcriptional level, which is the most common mechanism for animal miRNAs.

To determine whether the negative regulatory effects of miR-143/145 on ERBB3 expression were mediated through the binding of miR-143/145 to the presumed sites in the 3’-UTR of the ERBB3 mRNA, the full length 3’-UTR of ERBB3 containing the two presumed miR-143/145 binding sites was placed downstream of the firefly luciferase gene in a reporter plasmid. The resulting plasmid was transfected into MCF-7 cells along with a transfection control plasmid (β-gal) and either pre-miR-143/145, anti-miR-143/145 or scrambled negative control RNAs. As expected, the luciferase activity was markedly reduced in cells transfected with pre-miR-143 or pre-miR-145, and the potency was increased when pre-miR-143 and pre-miR-145 were used in combination (Figure 2E). In contrast, the luciferase activity was significantly increased in cells transfected with either anti-miR-143 or anti-miR-145, and a maximal increase in activity was observed when cells were transfected simultaneously with both anti-miR-143 and anti-miR-145 (Figure 2E). Furthermore, we introduced point mutations into the corresponding complementary sites in the 3’-UTR of ERBB3 to eliminate the predicted miR-143/145 binding sites. This mutated luciferase reporter was unaffected by either overexpression or knockdown of miR-143/145 (Figure 2E). This finding suggested that the miRNA binding sites strongly contributed to the miRNA:mRNA interaction that mediated the post-transcriptional repression of ERBB3 expression. Interestingly, co-treatment with both miR-143 and miR-145 at the same time suppressed the luciferase reporter containing the miR-143/145–responsive 3’-UTR to a greater extent than when either miR-143 or miR-145 was introduced alone. In conclusion, our results demonstrated that miR-143/145 directly recognized and bound to the 3’-UTR of the ERBB3 mRNA transcript and synergistically suppressed ERBB3 expression in breast cancer cells.

### Co-treatment with miR-143 and miR-145 synergistically suppress proliferation and invasion in breast cancer cells

**Figure 3:**
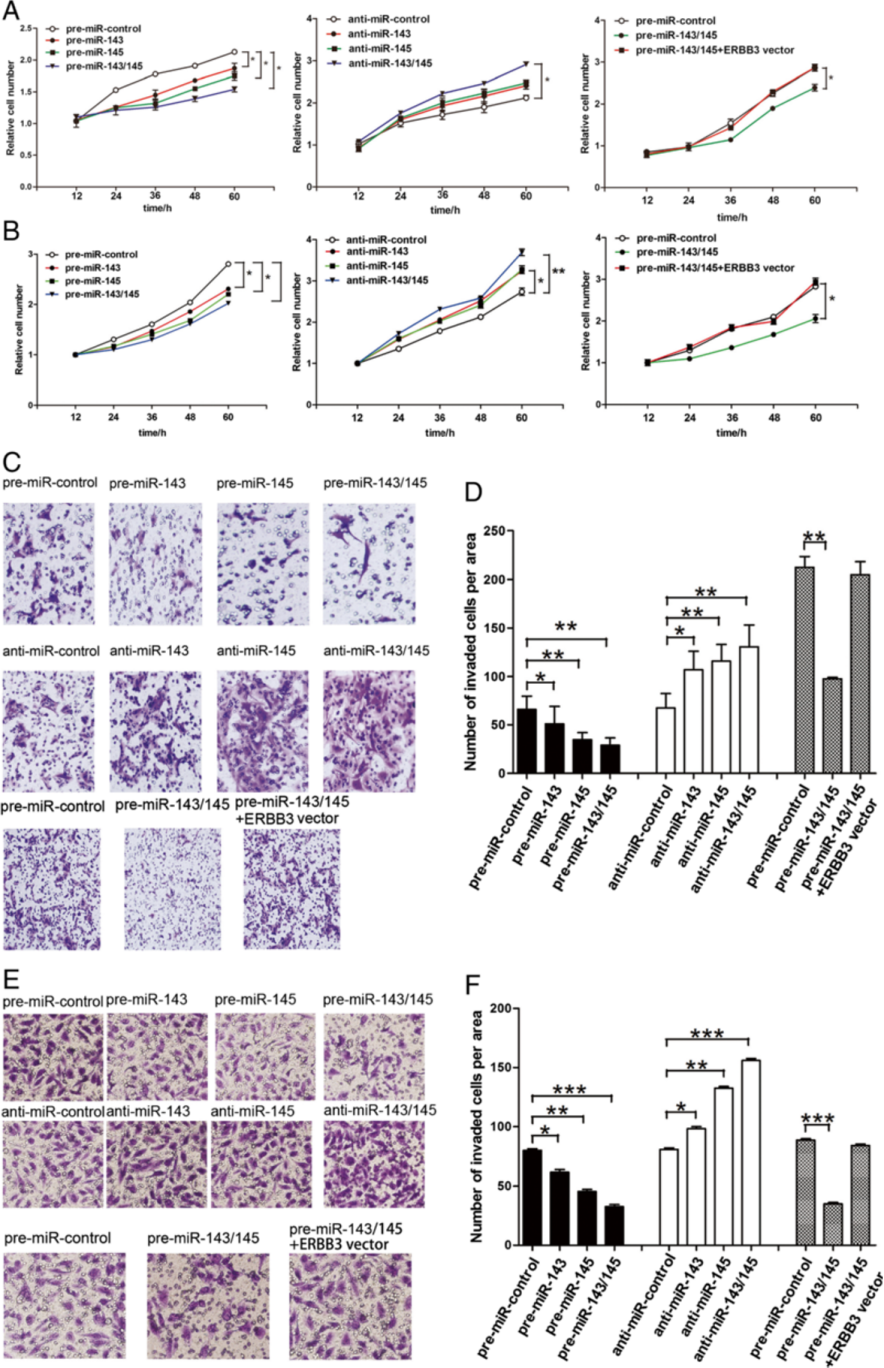
**miR-143/145 regulate proliferation and invasion of breast cancer cells by targeting ERBB3. (A and B)** MTT viability assays were performed 12, 24, 36, 48 and 60 h after the transfection of MCF-7 **(A)** and MBA-MD-231 **(B)** cells with pre-miR-control, pre-miR-143, pre-miR-145 or pre-miR-143/145 (left panel), or with anti-miR-control, anti-miR-143, anti-miR-145 or anti-miR-143/145 (middle panel), or with either pre-miR-control, pre-miR-143/145 or both pre-miR-143/145 and the ERBB3 overexpression vector (right panel). **(C-F)** Transwell invasion assays were performed after the transfection of MCF-7 **(C and D)** and MBA-MD-231 **(E and F)** cells with pre-miR-control, pre-miR-143, pre-miR-145 or pre-miR-143/145 (upper panel), or with anti-miR-control, anti-miR-143, anti-miR-145 or anti-miR-143/145 (middle panel), or with either pre-miR-control, pre-miR-143/145 or both pre-miR-143/145 and the ERBB3 overexpression vector (lower panel). **C** and **E**: representative image; **D** and **F**: quantitative analysis. * P < 0.05; ** P < 0.01.

We next analyzed the biological consequences of the decreased expression of miR-143 and miR-145 in breast cancer cells. We evaluated the effects of miR-143 and miR-145 on the proliferation and invasion of MCF-7 and MBA-MD-231 cells using the MTT and the Transwell invasion assays. In support of the notion that miR-143 and miR-145 function as tumor suppressive miRNAs [9], MCF-7 and MBA-MD-231 cells transfected with pre-miR-143/145 showed decreased proliferation; in contrast, knockdown of miR-143/145 had the opposite effect on cell proliferation (Figure 3A and B; left and middle panel). The Transwell invasion assay showed that the percentage of invaded cells was significantly lower in MCF-7 and MBA-MD-231 cells transfected with pre-miR-143/145 and higher in cells transfected with anti-miR-143/145 (Figure 3C-F; upper and middle panel). Meanwhile, although miR-143 and miR-145 can individually suppress cell proliferation and invasion, the simultaneous introduction of both miR-143 and miR-145 showed a cooperative repression of cell proliferation and invasion compared to effects of either miR-143 or miR-145 alone. These results demonstrated that miR-143 and miR-145 synergistically suppress proliferation and invasion in breast cancer cells.

### miR-143 and miR-145 suppress proliferation and invasion of breast cancer cells by targeting ERBB3

We next analyzed the biological consequences of the miR-143/145-driven repression of ERBB3 expression in breast cancer cells. We first investigated whether the overexpression or knockdown of ERBB3 would have an impact on cell proliferation and invasion in MCF-7 and MBA-MD-231 cells. To knock down ERBB3, a siRNA targeting ERBB3 was designed and then transfected into MCF-7 and MBA-MD-231 cells. To overexpress ERBB3, an expression plasmid designed to specifically express the full-length ORF of ERBB3 without the miR-143/145-responsive 3’-UTR was also constructed and transfected into MCF-7 and MBA-MD-231 cells. Efficient knockdown and overexpression of ERBB3 in MCF-7 and MBA-MD-231 cells is shown in Additional file 1: Figure S2A, D, Figure S3A and D. Consistent with previous studies showing that ERBB3 functions as an oncogene [5–7], MCF-7 and MBA-MD-231 cells transfected with the ERBB3 overexpression plasmid proliferated at a significantly higher rate (Additional file 1: Figure S2E and Figure S3E), whereas siRNA-mediated knockdown of ERBB3 decreased proliferation (Additional file 1: Figure S2B and Figure S3B). Similarly, MCF-7 and MBA-MD-231 cells transfected with the ERBB3 overexpression plasmid showed a stimulation of cell invasion (Additional file 1: Figure S2F and Figure S3F), and knockdown of ERBB3 with siRNAs significantly inhibited cell invasion (Additional file 1: Figure S2C and Figure S3C). Moreover, compared to MCF-7 and MBA-MD-231 cells transfected with pre-miR-143/145, the cells transfected with pre-miR-143/145 and the ERBB3 overexpression plasmid exhibited significantly higher proliferation rates (Figure 3A and B; right panel), suggesting that miR-143/145–resistant ERBB3 rescued the suppression of ERBB3 by miR-143/145 and attenuated the anti-proliferative effect of miR-143/145. Likewise, when MCF-7 and MBA-MD-231 cells were simultaneously transfected with pre-miR-143/145 and the ERBB3 overexpression plasmid, ERBB3 dramatically rescued the suppressive effect of miR-143/145 on cell invasion (Figure 3C-F; lower panel). These results demonstrated that ERBB3 is crucial to the proliferation and invasion of breast cancer cells, and that miR-143/145 can inhibit these cell functions through the silencing of ERBB3.

### miR-143 and miR-145 decrease the growth rate of breast cancer cells in vivo

**Figure 4:**
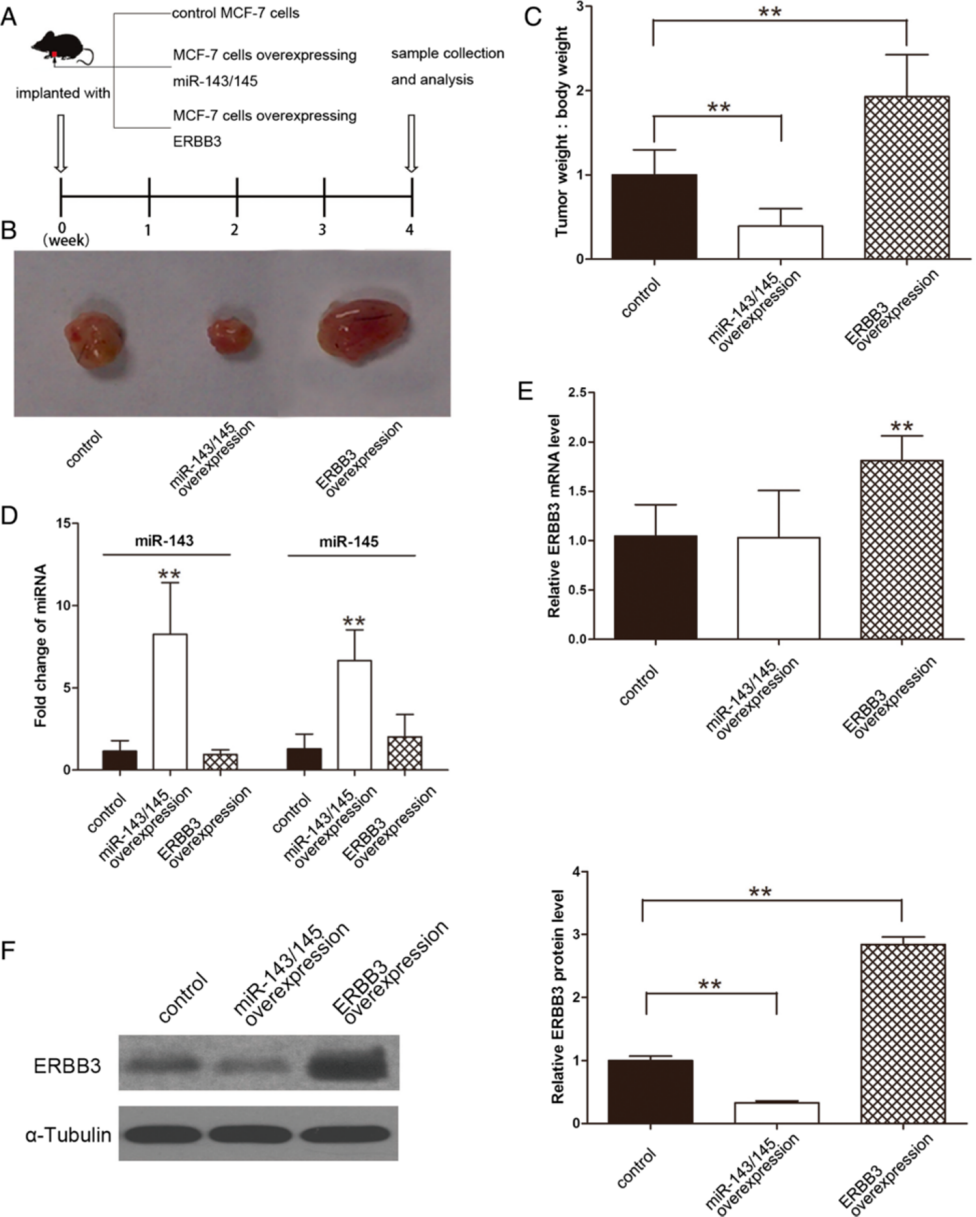
**Effects of overexpression of miR-143/145 or ERBB3 on the growth of breast cancer cell xenografts in mice. (A)** Flow chart of the experimental design. MCF-7 cells were either infected with a lentiviral expression vector to express miR-143 and miR-145 or transfected with an ERBB3 plasmid to overexpress ERBB3. MCF-7 cells (1 × 10^7^ cells per 0.1 mL) with increased miR-143/145 or ERBB3 levels were then implanted subcutaneously into 4-week-old C57/BL6 mice (7 mice per group), and tumor growth was evaluated at day 28 after cell implantation. **(B)** Representative image of the tumors from mice implanted with the control MCF-7 cells, the miR-143/145-overexpressing MCF-7 cells or the ERBB3-overexpressing MCF-7 cells. **(C)** Quantitative analysis of the tumor weights from mice implanted with control MCF-7 cells, miR-143/145-overexpressing MCF-7 cells or ERBB3-overexpressing MCF-7 cells. **(D)** Quantitative RT-PCR analysis of miR-143 and miR-145 levels in the tumors from the mice implanted with control MCF-7 cells, miR-143/145-overexpressing MCF-7 cells or ERBB3-overexpressing MCF-7 cells. **(E)** Quantitative RT-PCR analysis of ERBB3 mRNA levels in the tumors from mice implanted with control MCF-7 cells, miR-143/145-overexpressing MCF-7 cells or ERBB3-overexpressing MCF-7 cells. **(F)** Western blotting analysis of ERBB3 protein levels in the tumors from mice implanted with control MCF-7 cells, miR-143/145-overexpressing MCF-7 cells or ERBB3-overexpressing MCF-7 cells. Left panel: representative image; right panel: quantitative analysis. * P < 0.05; ** P < 0.01.

We next evaluated the effects of miR-143 and miR-145 overexpression on the growth of human breast cancer cell xenografts in mice. The use of viral constructs offered the possibility of rapidly producing high intracellular levels of mature miRNA via the endogenous miRNA processing pathway. To generate viral expression constructs, 300-bp fragments containing the genomic sequences of miR-143 and miR-145 were obtained by PCR amplification of human DNA and were then cloned into a lentiviral expressing vector. When MCF-7 cells were infected with these viral vectors, the expression of mature miR-143 and miR-145 was found to be 4–6-fold higher than the endogenous miRNA levels (Additional file 1: Figure S1E), and ERBB3 protein expression was significantly inhibited (Additional file 1: Figure S1F). MCF-7 cells were also transfected with an ERBB3 expression plasmid. MCF-7 cells (1 × 10^7^ cells per 0.1 mL) with elevated miR-143/145 or ERBB3 levels were implanted subcutaneously into 4-week-old C57/BL6 mice, and tumor growth was then evaluated at day 28 after cell implantation (Figure 4A). We observed a significant and marked reduction in the sizes and weights of the tumors in the miR-143/145-overexpressing group compared to control group, whereas the size and weight of the tumors in the ERBB3-overexpressing group was increased (Figure 4B and C). Total RNA was subsequently extracted from each xenograft and was used to evaluate the expression of mature miR-143 and miR-145. After 28 days of xenograft growth in vivo, tumors from the miR-143/145-overexpressing group showed a significant increase in the expression of mature miR-143 and miR-145 compared to tumors from the control group (Figure 4D). We also extracted total RNA and protein from each xenograft and evaluated the steady-state levels of ERBB3 by quantitative RT-PCR and Western blotting. Our results showed that ERBB3 mRNA levels were unchanged in the tumors from the miR-143/145-overexpressing group but increased in tumors from the ERBB3-overexpressing group (Figure 4E). Moreover, tumors from the miR-143/145-overexpressing group displayed reduced ERBB3 protein levels compared to tumors from the control group, whereas the tumors from the ERBB3-overexpressing group showed elevated ERBB3 protein levels (Figure 4F). These results were consistent with the findings of the in vitro assays, which firmly validated the biological role of miR-143/145 in breast tumorigenesis through the targeting of ERBB3.

## Discussion

Over the past decade, major advances in our comprehension of breast cancer biology have led to improved diagnostic and prognostic techniques and to the development of novel targeted therapies. However, the efficacy of new treatments remains limited by a combination of drug resistance and our limited understanding of tumor cell signaling pathways. Recently, an important role for the ERBB3 receptor in the genesis and progression of breast cancer has emerged [5–7]. ERBB3 is frequently overexpressed in breast cancer and tends to function as an oncogenic unit by forming heterodimers with ERBB2 [5–7]. Thus, ERBB3 offers a particularly promising molecular target for breast cancer therapy. However, very little is known about the regulation of ERBB3 expression in breast cancer. Thus, the molecular mechanisms underlying ERBB3 regulatory pathways need to be fully elucidated.

In this study, we predicted that ERBB3 is a target of miR-143 and miR-145, which are a cluster of miRNAs that have been reported in many studies to be downregulated and to function as tumor suppressors in most cancers [17, 18]. After measuring the expression levels of miR-143/145 and ERBB3 in human breast cancer tissue and paired noncancerous tissue, we detected an inverse correlation between miR-143/145 levels and ERBB3 protein levels. Furthermore, by overexpressing or knocking down miR-143/145 in MCF-7 cells, we experimentally validated that miR-143/145 directly inhibited ERBB3 translation. Finally, we showed that miR-143/145 inhibited ERBB3 expression and consequently promoted the proliferation and invasion of breast cancer cells, both in vitro and in vivo. The results delineate a novel regulatory network employing miR-143/145 and ERBB3 to fine-tune cell proliferation and invasion. We also provided evidence that restoration of ERBB3 expression can reverse miR-143/145-suppressed cell proliferation and invasion, suggesting that the targeting of ERBB3 is one mechanism by which the miR-143/145 cluster exerts its tumor suppressive function. Therefore, the modulation of ERBB3 by miR-143/145 may explain why the downregulation of miR-143/145 during breast carcinogenesis can promote cancer progression.

miRNAs can be grouped into families and clusters based on sequence and genomic location, respectively. miRNA family members are composed of multiple monocistronic miRNAs that have primary sequence similarity with the same seed sequence. Because the seed sequence contributes significantly to mRNA target specificity, miRNA family members are often thought to have extensive mRNA target overlap and redundant functions. On the other hand, miRNAs that are located nearby in the genome (within 10 kb) are considered to belonging to a single cluster. miRNA clusters are transcribed coordinately as polycistronic units that are processed to produce the individual members, which result in co-expression of the miRNAs. miRNAs originating from a single cluster often display corresponding sequence homology (e.g., miR-15a and miR-16) and therefore possess overlapping targets. However, for miRNAs in a cluster that do not share homology (e.g., miR-143 and miR-145), their individual and combined functionalities are less clear. One goal of this study was to determine whether miR-143 and miR-145 function individually or synergistically. In our study, bioinformatic analyses of the 3’-UTR of ERBB3 revealed two non-overlapping target elements for miR-143 and miR-145. Experimental validations supported the hypothesis that miR-143 and miR-145 exhibited a cooperative repression of ERBB3 expression and luciferase activity that was stronger than with either miR-143 or miR-145 alone. As a result, co-treatment with both miR-143 and miR-145 synergistically suppressed the growth and invasion of breast cancer cells compared with a single treatment with either miR-143 or miR-145. Because there are currently very few demonstrations of clusters of non-family miRNAs that target the same genes, our results provide novel evidence that individual miRNAs within a cluster that display a co-expression pattern but lack sequence homology can simultaneously and cooperatively repress a given target mRNA. Thus, although a single miRNA can clearly produce a phenotypic disturbance, when multiple miRNAs work in concert to repress a gene the effect may be more efficient and potent.

miRNAs are aberrantly expressed or mutated in cancer, which suggests a role for miRNAs as a novel class of oncogenes or tumor suppressor genes [14, 16]. Given the involvement of miRNAs in cancer development, the manipulation of cellular miRNA levels has emerged as a potential therapeutic strategy. Efforts to predictably alter intracellular transcript profiles by increasing specific miRNA levels through either transfection or viral delivery methods have demonstrated the potential of this strategy to modulate cellular physiology [28]. Conversely, attempts to reduce miRNA levels using biologically stable antisense oligonucleotides such as antagomirs have also proven capable of altering intracellular transcript profiles [29]. In this study, co-treatment with miR-143 and miR-145 showed a synergistic anti-tumor effect both in vitro and in vivo through the negative regulation of ERBB3 in human breast cancer. We hypothesize that a combination replacement treatment with both miR-143 and miR-145 will be a promising strategy for cancers showing downregulation of miR-143 and miR-145. In future studies, an effective drug delivery system needs to be developed for the application of miR-143 and miR-145 for breast cancer therapy.

Intense efforts have been made to inhibit the activities of the ERBB1/EGFR and ERBB2/HER2 receptors in cancer patients by designing antibodies against the ligand binding domains or small molecules against the tyrosine kinase domains. Both approaches have shown considerable clinical promise. However, increasing evidence suggests that the majority of cancer patients do not respond to these therapies, and those who do show an initial response ultimately become refractory to treatment [30]. ERBB3 has recently been proposed to be involved in the acquired resistance to ErbB-targeted drugs [30]. ERBB3 limits the impact of exclusively targeting ERBB1/EGFR or ERBB2/HER2 and provides a route for acquired resistance to anti-cancer drugs that inhibit ErbBs. Along with the biological impact of ERBB3 signaling on ERBB2-amplified breast cancer, active ERBB3 is a means of escape from therapeutic suppression by several tyrosine kinase inhibitors [30]. Thus, ERBB3 is likely to become a focus of breast cancer treatment. Recent studies have linked the acquisition of cancer drug resistance to the altered expression of miRNAs [31]. miR-143 and miR-145 have frequently been reported to be involved in drug resistance in many cancer types, although little evidence has been detected in breast cancer. Future studies are required to investigate whether miR-143 and miR-145 are involved in the mechanism of resistance to ErbB-targeted drugs in breast cancer. Because the targeting of ERBB3 has the potential to enhance the efficacy of other RTK inhibitors, combinatory treatments with both miR-143/145 and ErbB-targeted drugs may offer a viable strategy for breast cancer therapy. Future studies are necessary to address the clinical feasibility of implementing miRNA-based approaches to modulate sensitivity to chemotherapeutic agents.

Taken together, our results not only reveal a critical role for miR-143 and miR-145 as tumor suppressors in breast carcinogenesis through repression of ERBB3 translation but also show that different miRNAs within a cluster can simultaneously and cooperatively repress a given target mRNA. This study may provide a potential new target for future breast cancer therapy.

## Conclusions

Taken together, our results not only reveal a critical role for miR-143 and miR-145 as tumor suppressors in breast carcinogenesis through repression of ERBB3 translation but also show that different miRNAs within a cluster can simultaneously and cooperatively repress a given target mRNA. This study may provide a potential new target for future breast cancer therapy.

## Electronic supplementary material

Additional file 1: Table S1: Patients’ characteristics. **Figure S1.** The levels of miR-143/145 in breast cancer cells transfected with synthetic RNA oligonucleotides or infected with lentivirus. **Figure S2.** Effects of ERBB3 on proliferation and invasion of MCF-7 cells. **Figure S3.** Effects of ERBB3 on proliferation and invasion of MBA-MD-231 cells. (DOC 3 MB)

## Abbreviations

miRNA: microRNA
mRNA: Messenger RNA
MAPK: Mitogen-activated protein kinase
PI3K: Phosphoinositide 3-kinase
DMEM: Dulbecco’s modified Eagle medium
FBS: Fetal bovine serum
cDNA: Complementary DNA
CT: Circle threshold
PCR: Polymerase chain reaction
qRT-PCR: Quantitative real-time polymerase chain reaction
RT: Reverse transcription
Sec: Second
min: Minute
h: Hour
snRNA: small nuclear RNA
NC: Normal control
Bp: Base pair
Opti-MEM: A modification of Eagle’s minimum essential media
ORF: Open reading frame
siRNA: Small interference RNA
UTR: Untranslated regions
MTT: 3-(4,5-Dimethylthiazol-2-yl)-2,5-diphenyltetrazolium bromide
PAGE: Ployacrylamine gel electrophoresis
PBS: Phosphate buffer saline
PI: Propidium Iodide
PMSF: Phenylmethanesulfonyl fluoride
PVDF: Polyvinylidene fluoride
SDS: Sodium dodecyl sulfate
TBS: Tris buffered saline
TBST: Tris buffered saline and Tween
Tris: Hydroxymethyl aminoethane
TEMED: Tetramethylethylenediamine
Acryl: Acrylamide
APS: Ammonium persulfate
BCA: Bicinchoninic acid
DEPC: Diethyl Pyrocarbonate
DMSO: Dimethyl sulfoxide
ECL: Enhanced chemiluminescene
HCl: Hydrochloric acid.
