# Supplementary material for: miR-143 and miR-145 synergistically regulate ERBB3 to suppress cell proliferation and invasion in breast cancer
Source: Mol Cancer. 2014 Sep 24;13:220. doi: 10.1186/1476-4598-13-220 (PMC4181414; doi:10.1186/1476-4598-13-220)

**Supporting online material**

**miR-143 and miR-145 synergistically regulate ERBB3 to suppress cell proliferation and invasion in breast cancer**

**Additional file 1 Table S1.** Patients’ Characteristics

| Patients’ characteristics | | | | |  |
| --- | --- | --- | --- | --- | --- |
| Case No. | Clinical History | Gender | Age (years) | TNM Stage | HER-2 Status |
| BC #1 | IDC | Female | 44 | II-III | + |
| BC #2 | IDC | Female | 60 | II | - |
| BC #3 | IDC | Female | 47 | II-III | + |
| BC #4 | IDC | Female | 49 | II-III | + |
| BC #5 | IDC | Female | 46 | I-II | - |
| BC #6 | IDC | Female | 48 | III | - |

**Additional file 1 Figure S1. (A and B)** Quantitative RT-PCR analysis of miR-143 (A) and miR-145 (B) levels in MCF-7 cells treated with pre-miR-control, pre-miR-143 or both pre-miR-143 and pre-miR-145 and in cells treated with anti-miR-control, anti-miR-143 or both anti-miR-143 and anti-miR-145. **(C and D)** Quantitative RT-PCR analysis of miR-143 (C) and miR-145 (D) levels in MBA-MD-231 cells treated with pre-miR-control, pre-miR-143 or both pre-miR-143 and pre-miR-145 and in cells treated with anti-miR-control, anti-miR-143 or both anti-miR-143 and anti-miR-145. **(E)** Quantitative RT-PCR analysis of miR-143 and miR-145 levels in MCF-7 cells infected with a lentiviral miR-143/145 expressing vector. **(F)** Western blotting analysis of ERBB3 protein levels in MCF-7 cells infected with a lentiviral miR-143/145 expressing vector. Left panel: representative image; right panel: quantitative analysis.* P < 0.05; ** P < 0.01.


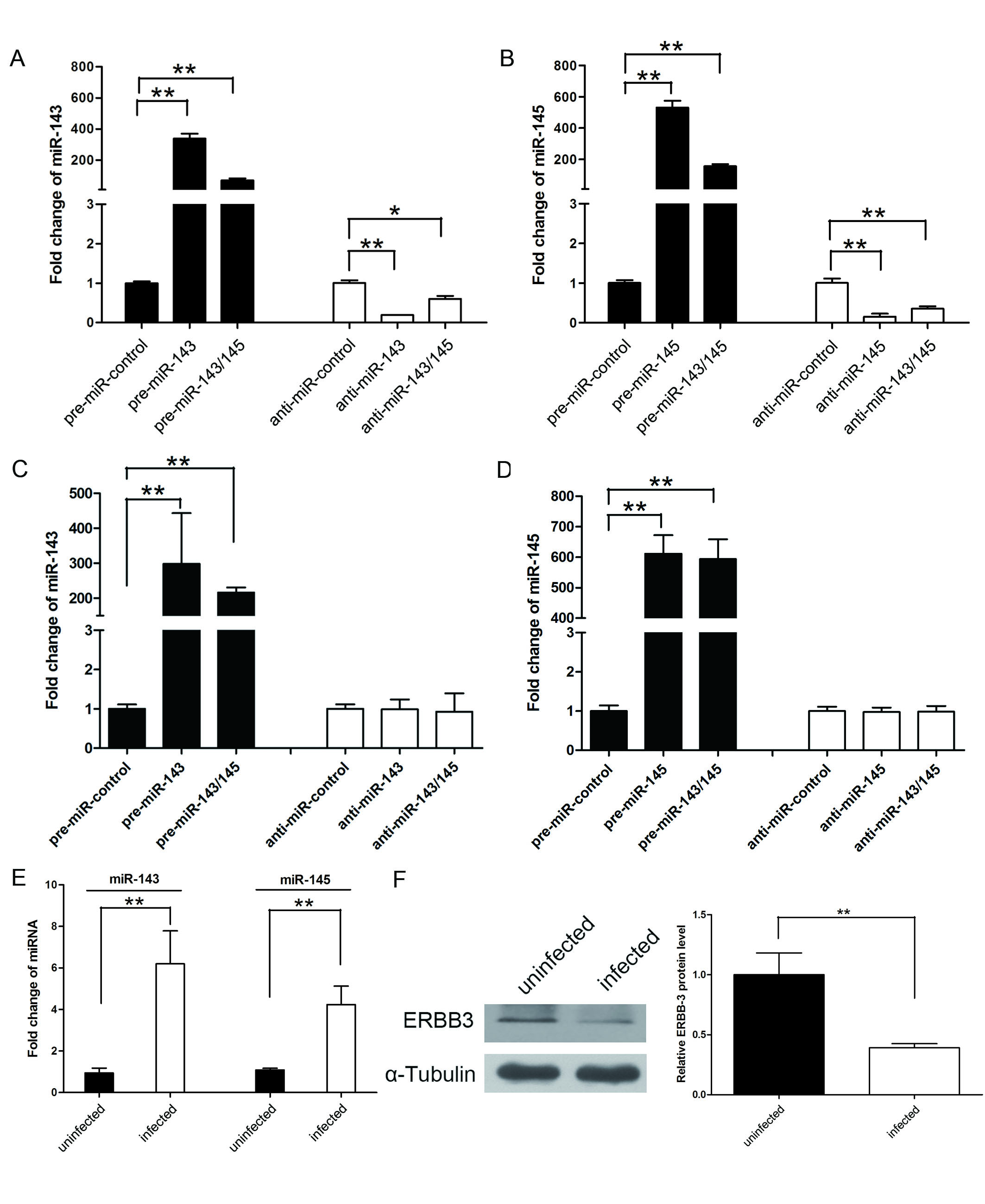


**Additional file 1 Figure S2. Effects of ERBB3 on proliferation and invasion of MCF-7 cells. (A)** Western blot analysis of ERBB3 protein levels in MCF-7 cells treated with either a scrambled control siRNA or an ERBB3 siRNA. Upper panel: representative image; lower panel: quantitative analysis. **(B)** MTT viability assays were performed 12, 24, 36, 48 and 60 h after the transfection of MCF-7 cells with either a scrambled control siRNA or an ERBB3 siRNA. **(C)** Transwell invasion assays were performed after the transfection of MCF-7 cells with either a scrambled control siRNA or an ERBB3 siRNA. Upper panel: representative image; lower panel: quantitative analysis. **(D)** Western blot analysis of ERBB3 protein levels in MCF-7 cells treated with either a control vector or an ERBB3 overexpression vector. Upper panel: representative image; lower panel: quantitative analysis. **(E)** MTT viability assays were performed 12, 24, 36, 48 and 60 h after the transfection of MCF-7 cells with either a control vector or an ERBB3 overexpression vector. **(F)** Transwell invasion assays were performed after the transfection of MCF-7 cells with either a control vector or an ERBB3 overexpression vector. Upper panel: representative image; lower panel: quantitative analysis. * P < 0.05; ** P < 0.01.

**
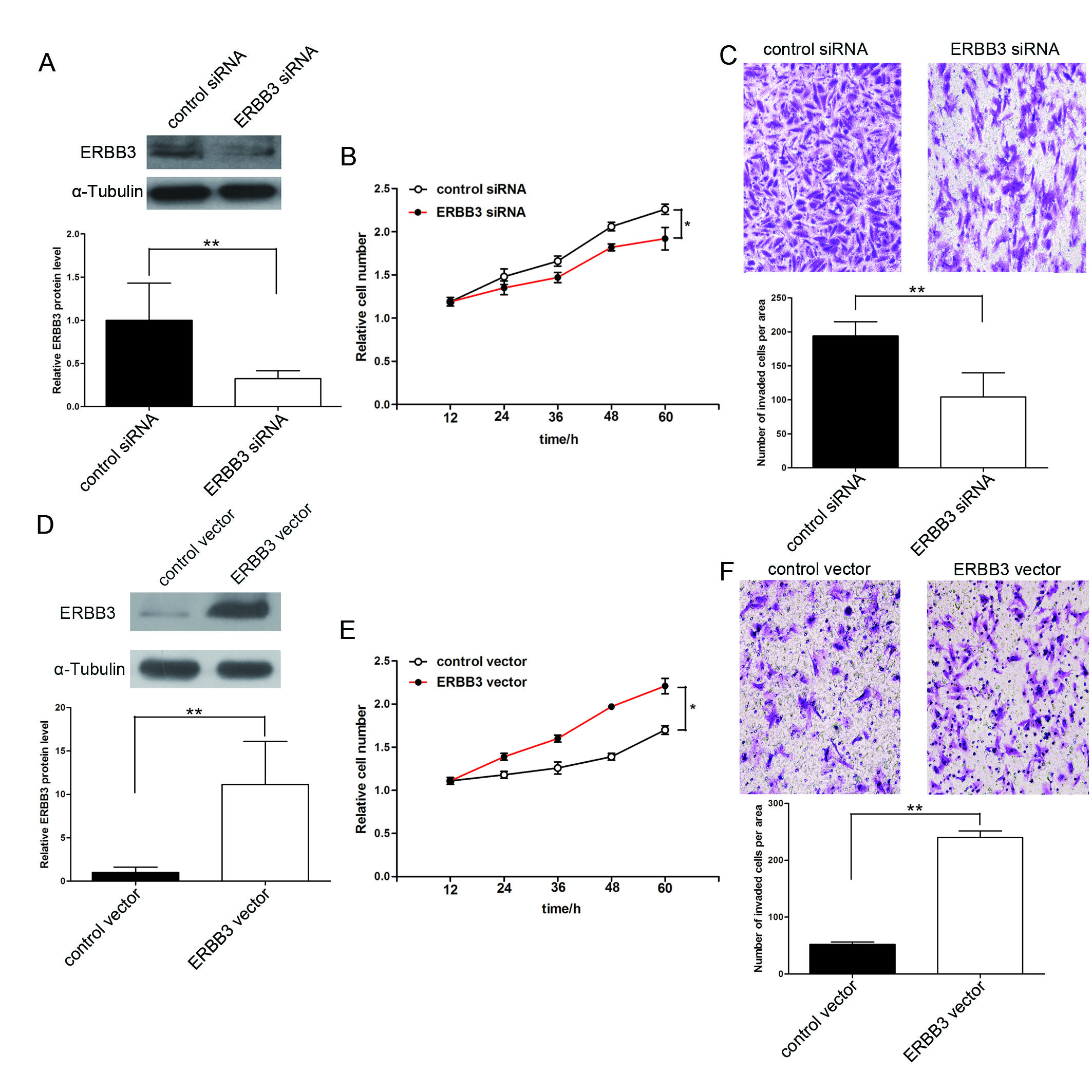
**

**Additional file 1 Figure S3. Effects of ERBB3 on proliferation and invasion of MBA-MD-231 cells. (A)** Western blot analysis of ERBB3 protein levels in MBA-MD-231 cells treated with either a scrambled control siRNA or an ERBB3 siRNA. Upper panel: representative image; lower panel: quantitative analysis. **(B)** MTT viability assays were performed 12, 24, 36, 48 and 60 h after the transfection of MBA-MD-231 cells with either a scrambled control siRNA or an ERBB3 siRNA. **(C)** Transwell invasion assays were performed after the transfection of MBA-MD-231 cells with either a scrambled control siRNA or an ERBB3 siRNA. Upper panel: representative image; lower panel: quantitative analysis. **(D)** Western blot analysis of ERBB3 protein levels in MBA-MD-231 cells treated with either a control vector or an ERBB3 overexpression vector. Upper panel: representative image; lower panel: quantitative analysis. **(E)** MTT viability assays were performed 12, 24, 36, 48 and 60 h after the transfection of MBA-MD-231 cells with either a control vector or an ERBB3 overexpression vector. **(F)** Transwell invasion assays were performed after the transfection of MBA-MD-231 cells with either a control vector or an ERBB3 overexpression vector. Upper panel: representative image; lower panel: quantitative analysis. * P < 0.05; ** P < 0.01.


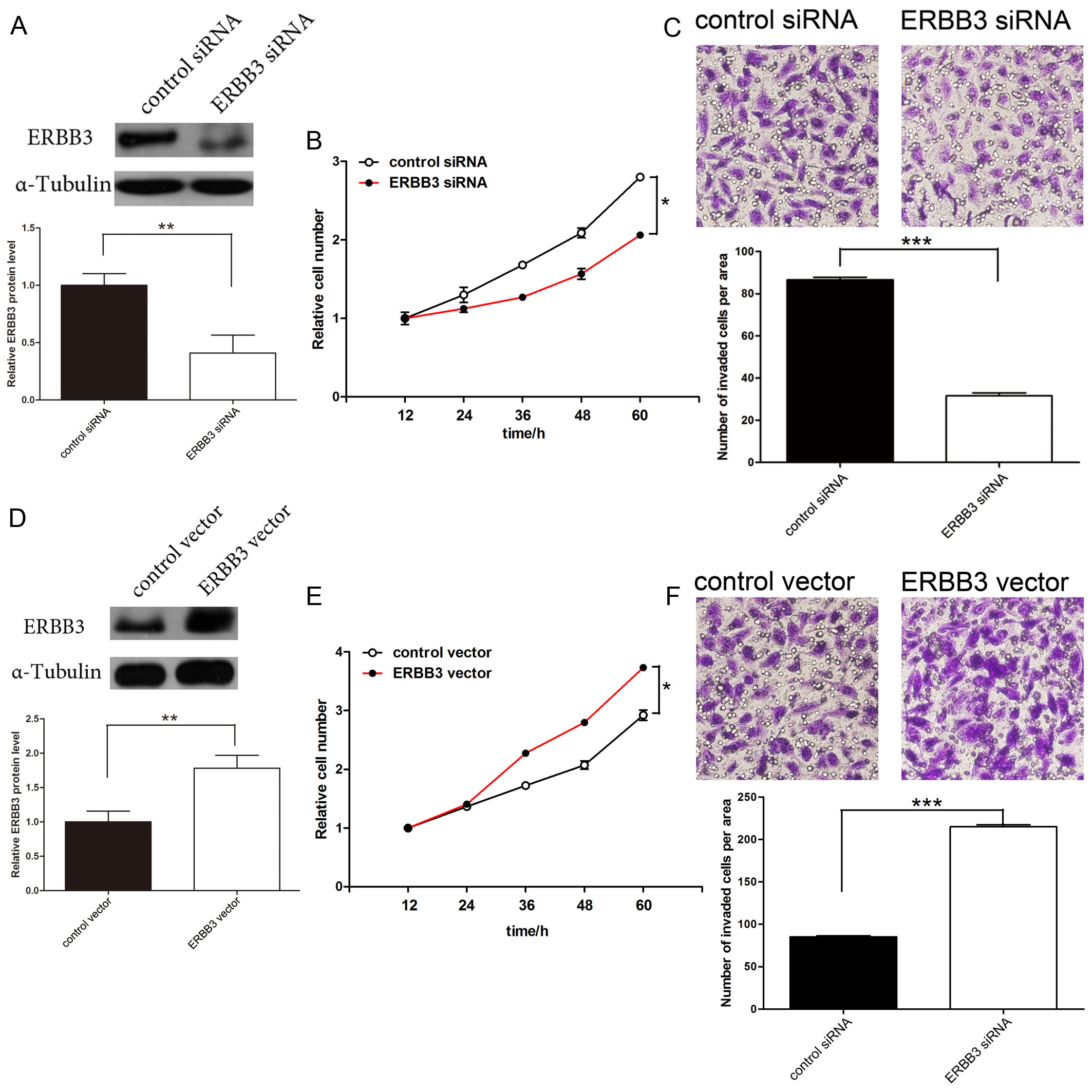

Supplement: Supplementary file 1 — Additional file 1: Table S1: Patients’ characteristics. Figure S1. The levels of miR-143/145 in breast cancer cells transfected with synthetic RNA oligonucleotides or infected with lentivirus. Figure S2. Effects of ERBB3 on proliferation and invasion of MCF-7 cells. Figure S3. Effects of ERBB3 on proliferation and invasion of MBA-MD-231 cells. (DOC 3 MB) [file 12943_2014_1422_MOESM1_ESM.doc]
